# Epoxy Cross-Linked Collagen and Collagen-Laminin Peptide Hydrogels as Corneal Substitutes

**DOI:** 10.3390/jfb4030162

**Published:** 2013-08-28

**Authors:** Li Buay Koh, Mohammad Mirazul Islam, Debbie Mitra, Christopher W. Noel, Kimberley Merrett, Silvia Odorcic, Per Fagerholm, William. Bruce Jackson, Bo Liedberg, Jaywant Phopase, May Griffith

**Affiliations:** 1 Integrative Regenerative Medicine Center, Department of Physics, Chemistry and Biology, Linköping University, SE 581 83 Linköping, Sweden; E-Mails: mayko@ifm.liu.se (L.B.K.); jayph@ifm.liu.se (J.P.); 2 Swedish Nanoscience Center, Karolinska Institute, 171 77 Stockholm , Sweden; E-Mail: mohammad.islam@ki.se; 3 Integrative Regenerative Medicine Center & Department of Clinical and Experimental Medicine, Cell Biology Building, Linköping University, SE 581 85 Linköping, Sweden; E-Mails: kim.merrett@sympatico.ca (K.M.); per.fagerholm@liu.se (P.F.); 4 Ottawa Hospital Research Institute, University of Ottawa Eye Institute, 501 Smyth Rd. Ottawa, ON K1H 8L6, Canada; E-Mails: debbiemitra@hotmail.com (D.M.); christophernoel90@gmail.com (C.W.N.); silvia.odorcic@gmail.com (S.O.); bjackson@ohri.ca (W.B.J.); 5 Center for Biomimetic Sensor Science, Nanyang Technological University, Singapore; E-Mail: bliedberg@ntu.edu.sg

**Keywords:** biomimetic materials, cross-linking, collagen, cornea, tissue engineering

## Abstract

A bi-functional epoxy-based cross-linker, 1,4-Butanediol diglycidyl ether (BDDGE), was investigated in the fabrication of collagen based corneal substitutes. Two synthetic strategies were explored in the preparation of the cross-linked collagen scaffolds. The lysine residues of Type 1 porcine collagen were directly cross-linked using l,4-Butanediol diglycidyl ether (BDDGE) under basic conditions at pH 11. Alternatively, under conventional methodology, using both BDDGE and 1-Ethyl-3-(3-dimethyl aminopropyl) carbodiimide (EDC)/N-hydroxysuccinimide (NHS) as cross-linkers, hydrogels were fabricated under acidic conditions. In this latter strategy, Cu(BF_4_)_2_·XH_2_O was used to catalyze the formation of secondary amine bonds. To date, we have demonstrated that both methods of chemical cross-linking improved the elasticity and tensile strength of the collagen implants. Differential scanning calorimetry and biocompatibility studies indicate comparable, and in some cases, enhanced properties compared to that of the EDC/NHS controls. *In vitro* studies showed that human corneal epithelial cells and neuronal progenitor cell lines proliferated on these hydrogels. In addition, improvement of cell proliferation on the surfaces of the materials was observed when neurite promoting laminin epitope, IKVAV, and adhesion peptide, YIGSR, were incorporated. However, the elasticity decreased with peptide incorporation and will require further optimization. Nevertheless, we have shown that epoxy cross-linkers should be further explored in the fabrication of collagen-based hydrogels, as alternatives to or in conjunction with carbodiimide cross-linkers.

## 1. Introduction

Corneal disease leading to vision loss is a major cause of blindness worldwide and a World Health Organization priority disease, particularly in the developing world [1]. While many causes of corneal blindness are treatable by donor human corneal transplantation, there is a severe shortage of high quality donor tissues despite innovations such as split grafts [2] and the use of gamma sterilization techniques to allow for processing of otherwise suboptimal tissue implants. Hence, there has been a range of technologies under development to augment and possibly replace the need for donor corneas, from fully cell-based injections of stem cells to promote regeneration [3] to the fabrication of biomaterials as prosthetic replacements that may be enhanced to enable corneal tissue regeneration [4].

We have now shown that biomimetic collagen-based materials that exhibit characteristics similar to that of the native extracellular matrix (ECM) can be developed as scaffolds to enable regeneration of the human cornea through recruitment of endogenous progenitor cells. Collagen is the most abundant ECM constituent in the body and contains the molecular recognition elements needed to elicit cellular responses and potentially carry out functions normally innate to that of the native tissue [5,6,7]. In a Phase I clinical study, 1-Ethyl-3-(3-dimethyl aminopropyl) carbodiimide (EDC) cross-linked recombinant human collagen (RHC) hydrogels were fabricated into corneal implants and grafted into 10 patients [8]. Two-year post-operative results showed that all 10 patients had regenerated their epithelium, stroma, and nerves, leading to restoration of the tear film and touch sensitivity in all corneas. At three years, post-operative, the corneas remained stable without immunosuppression.

However, these early implants were susceptible to microcrack formation and shearing [8]. Hence, in our clinical study, overlying sutures were used to retain the implants. The tight sutures crossing at the center of the cornea unfortunately impeded epithelial overgrowth on the implant toward the center, creating an ulcer-like condition that resulted in astigmatism as well as haze and graft thinning. Implants that were more elastic and amenable to the use of interrupted or continuous sutures that are placed peripherally without crossing the central cornea would have circumvented the suture-induced problem and given a better visual outcome. A new cross-linking strategy was therefore needed to fabricate implants of a higher elasticity. The incorporation of epoxy-based cross-linkers into collagen has been shown to enhance its mechanical properties. This was dependent upon coupling time, pH, and concentration of the cross-linking agent [9,10,11]. The bi-functional epoxy-based chemical cross-linker, l,4-Butanediol diglycidyl ether (BDDGE), in particular, has been found to produce materials with high tensile strength and improved elasticity under various conditions [11]. There are several ways in which BDDGE may be introduced into collagen materials, such as through direct cross-linking methods, by post-treatment of extracted collagen tissue or treatment of pre-cross-linked collagen material [12,13]. Typically, BDDGE is successively incorporated into pre-treated collagen tissue, followed by addition of other cross-linkers such as glutaradehyde or EDC [14]. Very recently, studies employing the epoxy-based BDDGE cross-linking of hydrogels have been reported [15,16].

**Scheme 1:**
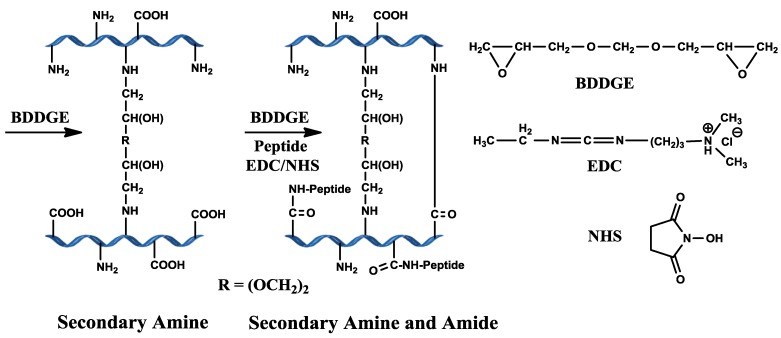
Scheme illustrating the predicted collagen cross-linking using BDDGE and EDC/NHS coupling.

Cross-linking of bi-functional BDDGE is pH dependent and occurs through secondary amine bond formation via epoxide ring opening by amine groups of collagen under basic pH conditions (Scheme 1). We examined BDDGE both as a stand-alone cross-linker and together with the use of EDC/ NHS sequential to BDDGE in the development of a potential corneal implant with high elasticity. 

Our earlier clinical trial results demonstrated that stroma in-growth to the central portions of the EDC based cross-linked implants took between 12 and 18 months [8], and nerve touch sensitivity was below that of healthy corneas. Thus, in addition to improving the mechanical properties, we also explored methods for enhancing cell and nerve in-growth into our BDDGE cross-linked collagen-based corneal implants.

To enhance both cell and nerve in-growth, we specifically examined the incorporation of two different laminin peptides, YIGSR and IKVAV, into the collagen hydrogel via EDC/NHS and BDDGE cross-linking. Laminin is a glycoprotein comprised of three polypeptide chains: A (440 KDa), B1 (230 KDa) and B2 (220 KDa). It is present in the basement membranes of cells and directly influences cell growth, migration, and proliferation [17,18]. Various peptides on the laminin macromolecule have distinctive biological effects [19]. We have previously demonstrated that a hydrogel matrix incorporating YIGSR promoted rapid epithelial cell overgrowth and both stromal cell and neurite extension into the implants through an acrylamide backbone [20]. In this project, we incorporated YIGSR and IKVAV laminin peptides using EDC/NHS coupling, directly into cell-free collagen hydrogels cross-linked with BDDGE and examined their effect on corneal epithelial cell attachment and neurite outgrowth.

## 2. Results and Discussion

### 2.1. pH and BDDGE Cross-Linking

Very few reports have established a direct chemical cross-linking method of BDDGE to uncross-linked collagen materials [21]. We conducted a series of experiments to determine the feasibility of directly cross-linking collagen using BDDGE. We varied the pH, collagen concentration, and use of a follow-on cross-linker. At pH 5, 10% porcine type I collagen did not form hydrogels with BDDGE alone. Hydrogels were not successfully fabricated under acidic conditions of esterification (pH 5, MES buffer), with a 1:1 BDDGE solution to collagen. However cross-linking of porcine collagen was observed, when Cu (II) tetrafluoroborate catalyst (Cu(BF_4_)_2_·xH_2_O) was used at pH 5. This catalyst has been shown to catalyze the formation of secondary amine bonds when in the presence of an amine and epoxide [22]. Amine bonds provide greater stability to collagen based materials and may subsequently enhance the mechanical properties and resistance towards enzymatic degradation [11].

Additionally, doubly cross-linked hydrogels (referred to as hybrid hydrogels) were fabricated by cross-linking with BDDGE followed by EDC/NHS at pH 5, showed gelation within 72 h at room temperature. Optimal cross-linking was achieved at a BDDGE:coll-NH_2_ ratio of 1:1, with 30% Cu catalyst loading, followed by immediate addition of EDC/NHS (0.5:1:NH_2_). 

An alternative strategy for BDDGE cross-linking of collagen without a catalyst was also explored. The iso-electric point of the lysine residues is roughly between pKa 10 and 11 [23]. Cross-linking around this pKa should result in optimal coupling since a high population of the amino groups of the lysine residues would no longer be positively charged [22]. Indeed, we showed that direct chemical coupling of BDDGE to NH_2_-collagen (1:1) at pH 11 resulted in hydrogel formation within 12 h at room temperature. By comparison, control collagen hydrogels coupled with a 0.7 EDC/NHS equimolar ratio relative to the amine groups in the collagen fibers cross-linked within 10 minutes at room temperature. BDDGE cross-linking is markedly slower than EDC/NHS cross-linking. This, however, will allow for incorporation of a delivery system where careful, homogenous mixing is required.

### 2.2. Properties of BDDGE-Collagen-Hydrogels

The properties of 10% collagen hydrogels cross-linked under different pH conditions are summarized in Table 1.

**Table 1:** Properties of l,4-Butanediol diglycidyl ether (BDDGE) cross-linked with 10% w/w porcine Type I collagen hydrogels content. Data were run in triplicate (*n* = 3) and expressed as mean (relative standard error %) and repeated for three independent experiments.

Properties	Human cornea	Type 1 porcine collagen		
		Control EDC/NHS	BDDGE	BDDGE-EDC/NHS
**pH**	-	5	11	5
**Optical properties**				
**Refractive index**	1.37–1.38 [24]	1.35	1.35	1.35
**White light transmission (%)**	>85 [25]	82.1 (2.1%)	86 (1.3%)	86 (1.3%)
**Backscatter (%)**	6.0-8.0 [25]	2.8 (10.3%)	1.9 (6%)	0.4 (43%)
**Mechanical properties**				
**Tensile strength (MPa)**	3.8 [26]	0.19 (3%)	0.21 (2.7%)	0.44 (1.3%) *
**Elongation at break (%)**	-	23.13 (2%)	14.02 (0.7%) *	147 (15.7%) *
**Young** **’s modulus (MPa)**	3.0–13.0 [27]	1.88 (6.4%)	2.86 (1.6%) *	2.69 (2.8%) *
**Thermal stability**				
**Denaturation temperature (°C)**	65.1 [28]	46.8	52.9	53.6
**Water content (%)**	80 [29]	91	92	92

* Denotes significant difference (*p* < 0.05) when compared against the EDC/NHS control.

#### 2.2.1. Physical and Optical Properties

The optical clarity of the BDDGE cross-linked materials varied depending on the cross-linking conditions. The refractive index measured in both EDC and BDDGE coupled materials was 1.35, slightly lower than that of the human cornea, likely due in part to the absence of a highly ordered structure typical of the native stroma. The 10% w/w hydrogels cross-linked with BDDGE at pH 11 or at pH 5 (BDDGE-EDC/NHS) have a light transmission of 86%, comparable to that of the human cornea. In all cases, the backscatter measured was well below the range observed for that of the human cornea. 

The heating of type I collagen solutions and hydrogels leads to the denaturation of the native triple helix structure of collagen. DSC can readily measure this transition from triple helix into a single-chain random coil conformation. The denaturing transition occurs at a temperature (Td), which is dependent upon the nature and the degree of cross-linking. The introduction of covalent bonds (e.g., cross-links) is known to increase the stability of the triple helix structure and, thus, increase the temperature at which the denaturing transition occurs [30]. The Td for pH 11 BDDGE cross-linked hydrogel is 52.9 °C, lower than that of the human cornea at 65.1 °C [28] and five degrees higher compared to the EDC/NHS cross-linked hydrogels (Table 1). Cross-linking by both BDDGE and EDC effectively stabilized the hydrogels as the denaturation temperature of the resultant collagen hydrogels showed an increase in the Td value relative to a non-cross-linked collagen solution which has a denaturation temperature of 36.6 °C. The higher Td indicates that BDDGE cross-linking imparts improved thermal stability to the hydrogel compared to that of EDC cross-linked hydrogels as demonstrated by the BDDGE cross-linked hydrogels at both pH 5 and pH 11. 

#### 2.2.2. Mechanical Properties of BDDGE-Collagen Hydrogels

The tensile strength of collagen hydrogels cross-linked with BDDGE under basic conditions at pH 11 was 0.21 MPa, similar to that of the EDC/NHS cross-linked gels (Table 1). The BDDGE cross-linked hydrogels, however, were stiffer with Young’s modulus at 2.86 MPa compared to that of the EDC hydrogels at 1.88 MPa. Accordingly, they were slightly less elastic than the EDC cross-linked hydrogels. The stiffer BDDGE hydrogels were likely a result of the cross-linking being carried out close to the pKa of lysine at 10.4 [23], allowing a higher degree of binding of lysine residues to BDDGE. 

The EDC/NHS and BDDGE cross-linked 10% collagen hydrogels showed a higher tensile strength of 0.44 MPa, compared to the 0.21 MPa for the pH 11 BDDGE cross-linked hydrogels. In addition, these hydrogels were highly elastic, allowing for a 147% elongation at break. In most cases, materials with high elasticity have not been fabricated without compromising tensile strength values in methods where direct cross-linking is used [9,10,11]. 

### 2.3. *In Vitro* Biological Stability

**Figure 1:**
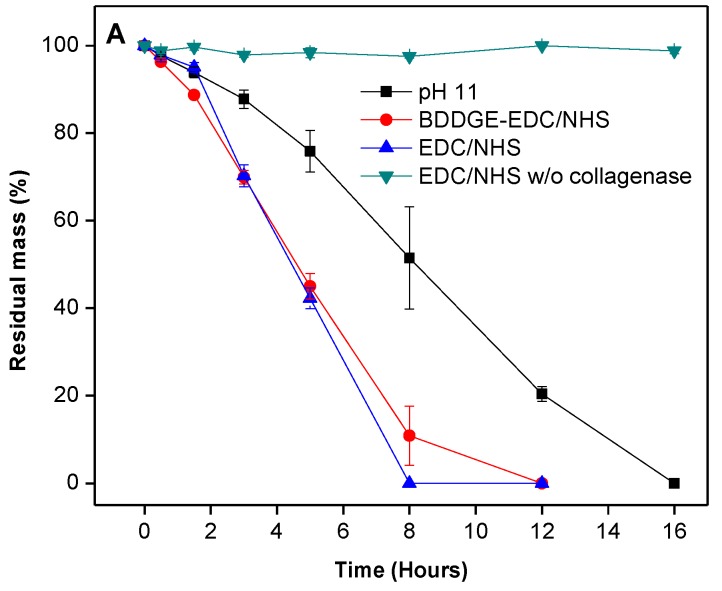
Degradation profile of 10% w/w BDDGE cross-linked hydrogels after exposure to collagenase and compared to EDC/NHS cross-linked hydrogels.

To assess biocompatibility, a degradation study was conducted to measure the relative resilience of BDDGE cross-linked hydrogels to collagenase degradation, compared to EDC/NHS hydrogel controls. The BDDGE cross-linked hydrogels (pH 11) took twice as long to be completely digested, *i.e*., 16 h compared to 8 h for EDC/NHS cross-linked hydrogels (Figure 1). The sequentially cross-linked collagen hydrogels demonstrated similar stability to the EDC/NHS controls. When interpreting these results, it is important to consider that the EDC and sequentially cross-linked hydrogels were formed at pH 5 whereas the BDDGE gels were synthesized at pH 11. Thus, the protein conformation likely would have been altered during the cross linking process and this may have had downstream effects on the collagenase degradation profiles. Additionally, the variability in collagenase resistance could, in part, be due to the chemical bonding. BDDGE facilitates an addition reaction between an epoxide group and lysine residue generating a secondary amine and hydroxyl group, whereas EDC activates the carboxylic acids on the collagen fibers towards amide formation.

### 2.4. Femtosecond Laser-Assisted Cuts

**Figure 2:**
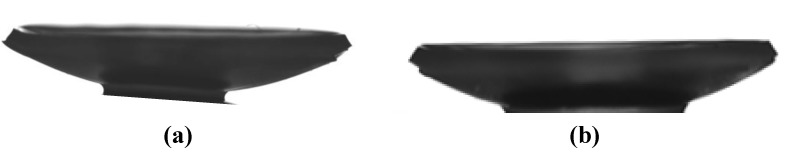
Femtosecond laser-assisted tophat cuts of the BDDGE cross-linked hydrogels that were cross-linked (**a**) at pH 11; and (**b**) at pH 5, with subsequent cross-linking with EDC/NHS.

To evaluate the ease of hydrogel handling and feasibility of creating customized cuts, three samples of each hydrogel underwent femtosecond laser-assisted cuts (IntraLase^TM^, Abbott Medical Optics, Inc., Abbott Park, IL, USA). This is becoming a commonly used technique in corneal refractive surgery. The femtosecond laser creates minimal collateral tissue damage but uses near infrared wavelength pulses to create precise and programmable cuts. In both the BDDGE pH 11 and BDDGE-EDC/NHS hydrogels, the femtosecond laser created well-defined trephine and tophat cuts (Figure 2). Preliminary experiments indicate that BDDGE coupled materials can be reliably and precisely cut with femtosecond laser given their relatively high tensile strength and appropriate elasticity.

### 2.5. Effects of addition of Laminin Peptides to BDDGE Cross-Linked Hydrogels

In order to fabricate implants that were sufficiently robust for future grafting as alternatives to donor corneal tissue, the collagen content was increased to 18%. This resulted in a slight decrease in light transmission. Mechanical and physical properties of the 18% hydrogels (Table 2) were similar to 10% hydrogels (Table 1), with the BDDGE- EDC/NHS hydrogels displaying more suitable mechanical properties to that of the BDDGE hydrogels. Increased tensile strength and elasticity are important in order to improve the surgical manipulability as these enhanced properties may facilitate the use of continuous and interrupted sutures within the hydrogel materials. 

**Figure 3:**
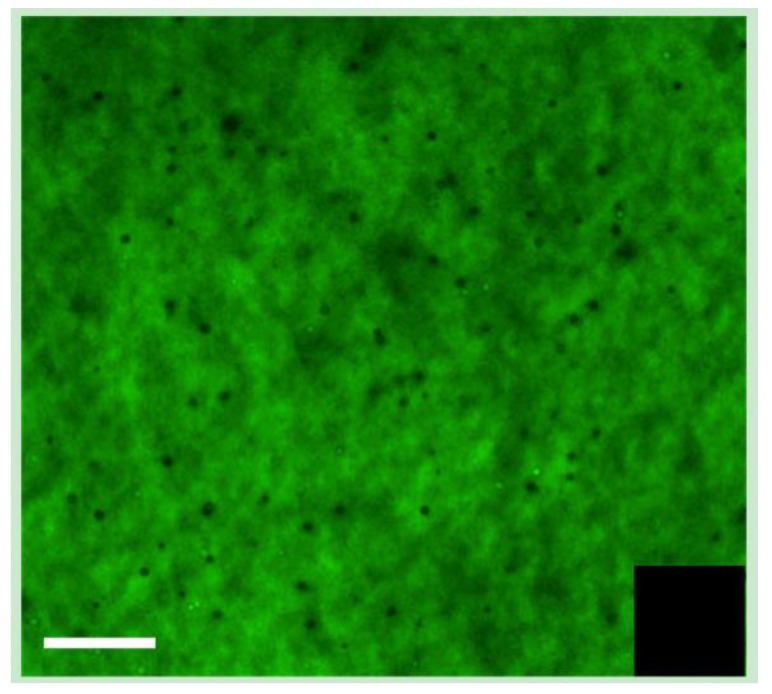
Visualization of biotin-IKVAV peptides incorporated into BDDGE-EDC/NHS collagen hydrogel, with FITC-streptavidin. The inset shows the EDC/NHS control hydrogel without peptide. Scale bar = 100 μm.

The incorporation of the laminin peptides, YIGSR, and IKVAV, into the collagen hydrogels was verified by immunofluorescence. For this purpose, biotinylated peptides were prepared and visualized using FITC-conjugated streptavidin following incorporation (Figure 3).

**Table 2:** Properties of BDDGE cross-linked, 18% w/w porcine Type I collagen hydrogels (*n* = 3 samples per group). Data are expressed as mean (relative standard error %) and repeated for three independent experiments.

Properties	Human cornea	Type 1 porcine collagen				
		Control EDC/NHS	BDDGE	BDDGE-EDC/NHS	YIGSR	IKVAV
**pH**	-	5	11	5	5	5
**Optical properties**						
**White light transmission (%)**	>85 [25]	85.67 (0.8%)	81.07 (0.7%)	84.17 (0.8%)	84.03 (1.3%)	82.10 (0.8%)
**Mechanical properties**						
**Tensile strength (MPa)**	3.8 [26]	0.12 (14.4%)	0.10 (17.3%)	0.16 (7.2%)	0.13 (4.4%)	0.17 (44%)
**Elongation at break (%)**	-	44.52 (24.4%)	16.63 (6.2%) *	120.48 (9.3%) *	23.81 (5.9%)	44.76 (2.6%)
**Young’s modulus (MPa)**	3.0-13.0 [27]	0.64 (39.6%)	1.21 (15.7%) *	0.21 (8.2%) *	0.98 (1.7%)	0.55 (46%)
**Thermal properties**						
**Denaturation temperature (°C)**	65.1 [28]	49.1	65.9	49.5	47.2	55.0 & 64.4

* Denotes significant difference (*p* < 0.05) when compared against the EDC/NHS benchmark.

*In Vitro* Biological Stability of Laminin Peptide-BDDGE-Collagen Hydrogels

**Figure 4:**
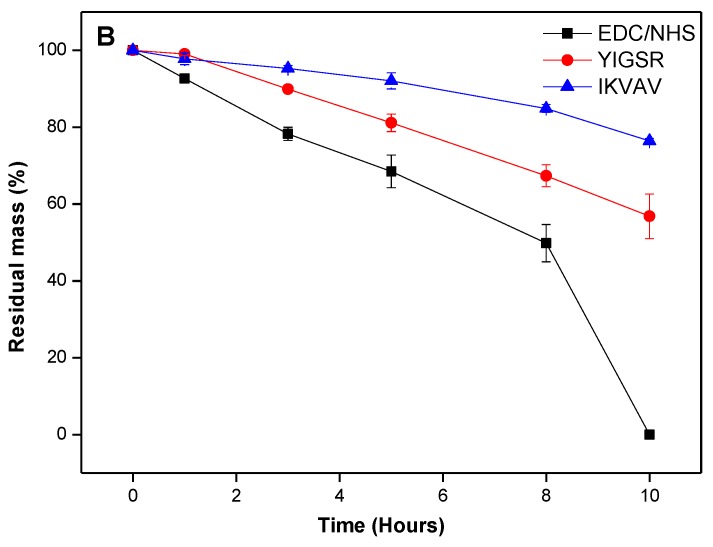
Degradation profile of 18% w/w BDDGE cross-linked hydrogels after exposure to collagenase and compared to EDC/NHS cross-linked hydrogels.

In the case of 18% w/w collagen hydrogels, increased stability was observed against collagenase due to incorporation of peptide, IKVAV and YIGSR (Figure 4). Residual mass was more than 50% in case of both peptide-incorporated hydrogels, which was significantly (*p* < 0.001) higher than the control hydrogel at 10 h. Although there was no significant difference in retention of residual mass in case of both IKVAV and YIGSR hydrogels after 10 h, IKVAV exhibited greater resistance. We reasoned that this might be due to the formation of additional cross-linking by IKVAV due to the number of lysine residues within the peptide. The greater overall stability of IKVAV containing hydrogels could be due to the formation of interpenetrating networks within the hydrogel, as shown by the two discrete Td values (Table 2). 

### 2.6. *In Vitro* Biocompatibility and Performance

**Figure 5:**
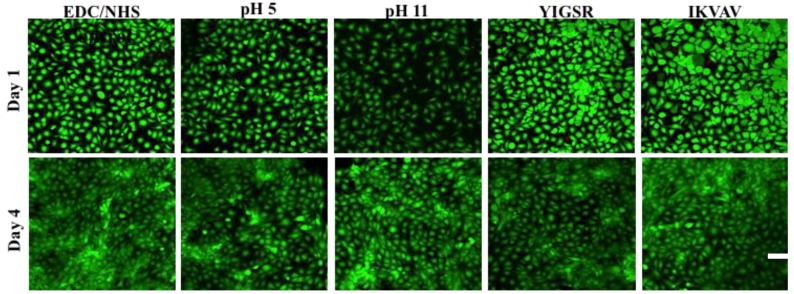
Confocal laser scanning microscope (CLSM) showing the biocompatibility of the BDDGE cross-linked hydrogels with negligible amount of dead HCECs from the live/dead stain at day one and four respectively. Green and red fluorescence indicated live and dead HCECs, respectively. Scale bar = 100 μm.

**Figure 6:**
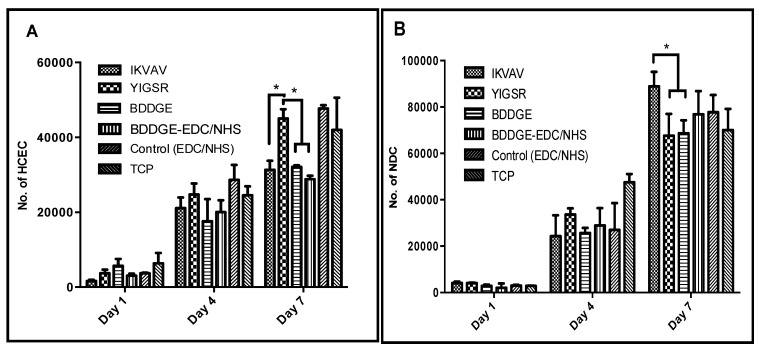
Proliferation rates of (**A**) human corneal epithelial cells; and (**B**) neuronal progenitor cells (NDC cell line) on different hydrogels at days one, four and seven. Samples were run in triplicate (*n* = 3) and results were expressed as means, and repeated for three independent experiments. * Statistical significance by ANOVA (*p* < 0.05).

It is important to evaluate the biocompatibility of the BDDGE coupled hydrogels as the clinically tested EDC cross-linked biomaterials were found to promote corneal cell and nerve regeneration in all our patients. Live/Dead staining of immortalized human corneal epithelial cells (HCECs) seeded on BDDGE cross-linked hydrogels fabricated under different conditions showed very low/no dead cells (Figure 5). This demonstrated that the hydrogels were non-cytotoxic. The cell adhesion and spread on the hydrogels was also morphologically distinct by microscopic examination as the cells were flattened and spread out on the hydrogels. The proliferation rate of HCECs and neuronal progenitors (derived from rodent dorsal root ganglia fused with neuroblastoma cells (NDCs)), on BDDGE cross-linked hydrogels were also examined. A MTS assay showed that all the hydrogels tested successfully supported HCEC proliferation, albeit at different rates (Figure 6). The differences in proliferation were most distinct by day seven of culture after pre-seeding on the 9 mm diameter hydrogels of 500 μm thickness. The rate of cell proliferation on BDDGE cross-linked hydrogels was slower than that on the EDC/NHS cross-linked hydrogels (Figure 6A). Addition of YIGSR peptide, however, increased the proliferation rate to the level of cells grown on EDC/NHS cross-linked hydrogels.

NDC proliferation was also supported by all the hydrogels. However, the highest proliferation rate was observed in the IKVAV collagen-hydrogels (Figure 6B).

Hydrogels with incorporated IKVAV and YIGSR had statistical significant influence (*p* < 0.05) on cell proliferation of NDCs and HCECs. By day seven of culture, the peptides were able to differentially affect cell growth. YIGSR enhanced HCEC growth while IKVAV enhanced neuronal cell growth. This was in keeping with previous reports in the literature. 

Although incorporation of IKVAV and YIGSR peptides enhanced neuronal and epithelial cell growth, the marked improvement in the mechanical properties of the hydrogels offered by the BDDGE cross-linking following by EDC/NHS enhancement was lost. Hydrogels showed properties that were similar to those of EDC/NHS cross-linked benchmarks only. This suggests a lack of incorporation of BDDGE coupling within the hydrogel matrix likely as a result of a competing reaction of the peptides and the BDDGE cross-linker present in solution during the time of gelation. The potential decreased BDDGE cross-linking would thus result in hydrogels that mainly consisted of EDC/NHS type coupling. These results, therefore, also show that modification of BDDGE-EDC/NHS hydrogels with peptide would be more effective with surface modifications instead of being incorporated into the bulk of the hydrogel. Further studies are required in order to test this hypothesis, by varying the experimental conditions such as the ratio of the coupling reagents; EDC, NHS, and BDDGE, as well as the manipulation of surface-conjugated peptides for optimized properties of the cross-linked collagen-based hydrogels.

## 3. Experimental Section

### 3.1. Materials

Porcine Type I collagen purchased from Sewon Cellontech (Seoul, South Korea) was lyophilized and reconstituted to make either a 10 w/w% or 18% w/w% solution. All other reagents were of analytical grade and used as received. 1-Ethyl-3-(3-dimethyl aminopropyl) carbodiimide (EDC) and 1,4-Butanediol diglycidyl ether (BDDGE) were both supplied by Sigma-Aldrich (Oakville, Ontario, Canada), Copper (II) Tetrafluoroborate Cu(BF_4_)_2_ was supplied by Strem Chemicals, (Newburyport, MA, USA) and N-hydroxysuccinimide (NHS) by Fluka (Buchs, Switzerland). Phosphate-buffered saline (PBS, pH 7.2) was prepared via tablets and obtained from Calbiochem Corp., (Darmstadt, Germany). Milli-Q deionized water was used throughout all necessary experiments. 

The peptides IKVAV and YIGSR-NH2 were synthesized on symphony automated peptide synthesizer (Protein Technologies Inc., Tucson, AZ, USA) using standard fluorenylmethoxycarbonyl (Fmoc) chemistry with HCTU (ChemPep Inc., Wellington, FL, USA) as the activating reagent. The synthesis was performed on a 0.1 mmol scale with Fmoc-Val-PEG-PS and Fmoc-PAL-PS resin (Applied Biosystems, Sweden) using a four-fold excess of amino acid in each coupling. The peptides were cleaved from the resin by treatment with a mixture of trifluoroacetic acid (TFA), and water (95:5 v/v; 10 mL per gram of polymer) for 2 h at room temperature. After filtration, TFA was evaporated and the peptides were precipitated by the addition of cold diethyl ether, centrifuged and lyophilized. The crude products were purified by reversed-phase HPLC on a semi-preparative C-18 column (Grace Vydac) and identified from MALDI-TOF spectra (Applied Biosystems Voyager DE-STR, Stockholm, Sweden).

### 3.2. Preparation of Collagen Cross-Linked Materials

0.4 mL aliquots of either 10 or 18 w/w% collagen were weighed out and mixed in a T-piece system, using a methodology similar to one we previously described [31]. For gels performed in basic conditions a buffer of 0.036 M Na_2_CO_3_ and 0.064 M NaHCO_3_ at pH 10 was used to fill the T-piece whereas a 0.625 MES buffer was used in acidic conditions. The collagen solution was adjusted to either pH 5 or 11 with microliter quantities of 2N aqueous NaOH, followed by thorough mixing. Calculated volumes of aqueous solutions of BDDGE, Cu(BF_4_)_2_, EDC and its co-reagent NHS were added to their respective solutions. For epoxide gels, the final “cross-linker-doped” collagen solution was mixed 150× at 4 °C whereas carbodiimide solutions were only capable of being mixed 50×. 

Laminin-derived cell-adhesive peptides were incorporated within the epoxide gels using the pH 5 formulations, with 0.001 equimolar of peptide relative to mole of amine in collagen. Additionally, biotinylated peptide and streptavidin was used to investigate the binding affinity between the peptides within the BDDGE hydrogel matrix. For incorporation of YIGSR and IKVAV peptides, a starting concentration of 18% w/w% collagen was used to allow for dilution of the collagen content. Confocal fluorescence microscopy (using a LSM700 confocal microscope, Carl Zeiss AB, Stockholm, Sweden) was used to examine the presence of biotinylated peptide inside the hydrogel. The final collagen solution was dispensed as flat sheet into glass molds, cured at 100% humidity at room temperature for 24 h, post-cured at 37 °C for 1 day, then washed extensively in PBS to remove any non-cross-linked substrate. Hydrogels cross-linked sequentially by both BDDGE and EDC/NHS were cured for 72 h.

### 3.3. Optical Property Measurements

Refractive indices of fully hydrated epoxide and carbodimide cross-linked hydrogels were recorded using an Abbe refractometer (Model C10, VEE GEE, Scientific Inc., Kirkland, WA, USA). The experiment was performed at 21 °C with bromonaphthalene as the calibration agent. Light transmission and back-scattering measurements were carried out at room temperature for white light (quartz-halogen lamp source) and for narrow spectral regions (centered at 450, 500, 550, 600, and 650 nm). Briefly, a custom-built instrument was used to measure the percent transmission of samples as compared to open beam intensity [30]. The relative percent of light back scattered from the collimated beam by the sample was measured with a circular array of 8 photodiodes, 30 degrees off axis.

### 3.4. Mechanical Property Measurements

The tensile strength, Young’s moduli and elongation at break of the hydrogels were determined on an Instron electromechanical universal tester (Model 3342) equipped with Series IX/S software, using a crosshead speed of 10 mm·min^−1^ and a gauge length for testing of 5 mm. Hydrogels with 0.55 mm thickness were equilibrated in PBS and cut into 10 mm × 5 mm rectangular sheets. A minimum of three specimens was measured for each hydrogel formulation and repeated for three independent experiments. 

### 3.5. Equilibrium Water Content Measurement

After removal from the molds, hydrogels were immersed in a PBS solution for 5 days at 4 °C. After removal of surface water through gentle blotting on filter paper, the samples were immediately weighed on a microbalance to record the “wet weight” (W_0_) of the sample. They were allowed to dry at room temperature under vacuum to constant weight (W). The total equilibrated water content of the hydrogels (W_t_) was calculated according to the following equation:

W_t_ = (W − W_0_)/W × 100% (1)

### 3.6. Thermal Properties: Differential Scanning Calorimetry (DSC)

The thermal properties of the hydrogels were tested using a Q2000 differential scanning calorimeter (TA Instruments, New Castle, DE). Heating scans were recorded within the range of 8 to 80 °C at a scan rate of 5 °C·min^−1^. Pre-weighed samples of the PBS-equilibrated hydrogels (weights ranging from 5 to 10 mg) were surface-dried with filter paper and hermetically sealed in an aluminum pan to prevent water evaporation. A resulting heat flux *versus* temperature curve was then used to calculate the denaturing temperature (T_d_). T_max_ of the endothermic peak gives the denaturing temperature. 

### 3.7. *In Vitro* Degradation

Briefly, hydrated cross-linked hydrogels (approximately 50 mg) were placed in vials containing 5 mL of a 5 U/mL collagenase in a PBS solution (Type I Collagenase from *Clostridium histolyticum*, 318 U/mg solid, Sigma-Aldrich, Oakville, ON, Canada), refreshed every 8 h. The vials were incubated in an oven at 37 °C. The gels were weighed at different time intervals after removal of surface water through blotting. The residual mass of the hydrogels was tracked as a function of time, relative to their initial hydrated weight. 

### 3.8. Femtosecond Laser-Assisted Cuts of Epoxide and Carbodiimide Cross-linked Hydrogels: Feasibility

Both hydrogels and cadaveric porcine control corneas underwent femtosecond laser-assisted trephine (cylindrical) and tophat cuts. Cylindrical cuts were programmed to span the entire depth of the hydrogel at a cut angle of 90 degrees, while the tophat cut was programmed with 90 degree anterior and posterior side cut angles. The dimensions, shape, and side cut angles were then captured for analysis using an optical profilometry system developed by Rejean Munger and David Priest at the Ottawa Hospital, Ottawa, Ontario, Canada. Optical profilometry captures silhouette images of cut hydrogels using a high-resolution camera positioned against a backlight. These images are subsequently analyzed using ImageJ Software (Java-based image processing, NIH freeware) to calculate their precise dimensions and side cut angles.

### 3.9. *In Vitro* Biocompatibility and Performance

To observe the biological effect of the different hydrogels, immortalized human corneal epithelial cells (HCEC) [32] and rodent hybrid dorsal root ganglia-neuroblastoma cells (NDC) [33] were seeded directly on the hydrogels. For cell culture, hydrogels of 6 mm diameter size were cut and placed in 24-well plate followed by sterilization with 1% v/v chloroform and 3X antibiotic solution consisting of 300 unit/mL penicillin and 300 µg/mL streptomycin. HCEC and NDC were seeded at 5 × 10^4^ cells/well respectively with a small volume of medium (50 µL) on the top of the hydrogel and incubated for 30 min. HCEC and NDC were maintained in EpiGRO™ medium (Millipore) and DMEM supplemented with 10% FCS and 1% penicillin and spectinomycin, respectively. Medium was replaced on alternative days and culture was maintained for 7 days. 

For both HCEC and NDC, proliferation was measured by the colorimetric MTS assay (CellTiter 96 Aqueous One solution, Promega, Madison, WI). Dehydrogenase enzymes from mitochondria within live cells convert the yellow tetrazolium salt [3-(4,5-dimethylthiazol-2-yl)-5-(3carboxymethoxyphenyl)-2(4-sulfophenyl)-2H-tetrazolium] to purple formazancrystals. Herein, the amount of formazan crystal formation is directly proportional to the number of live cells. The MTS assay was performed on days 1, 4 and 7 of cell culture. Hydrogels were rinsed three times in PBS and subsequently transferred into new wells prior to MTS assay to avoid the absorbance from any cells that may have been growing on the tissue culture plastic beneath the hydrogels. Subsequently, 20% v/v of MTS reagent in cell culture medium was added and cultures were incubated further for 4 h at 37 °C. Absorbance was measured at 490 nm using a spectrophotometric microplate reader (VERSA Max Microplate Reader, Molecular Device, Sunnyvale, CA, USA).

The biocompatibility of the different hydrogel formulations with HCEC survival was examined by using live/dead staining on day 1 and day 4 of culture. Calceinacetoxymethyl (Calcein AM) and ethidium homodimer-1 (EthD-1) (Invitrogen) were used to determine the viability of cells on the hydrogels. The concentration of Calcein AM and EthD-1staining solution were 2 µM and 4 µM respectively. The fluorescence images were visualized under fluorescent confocal microscope (LSM700, Carl Zeiss). Morphological inspection of the cells for flattening and spread (as opposed to rounded cells) gave an indication of the ability of each hydrogel formulation to support cell adhesion and spread.

Cell proliferation studies on hydrogels were repeated separately for three times and run in triplicate (*n* = 3). Data obtained from the different experiments are presented as mean values. Statistical significance of differences between two hydrogels cell proliferation was analyzed using single factor analysis of variance (ANOVA).

## 4. Conclusions

The BDDGE hydrogels exhibit high tailorability, given that the mechanical properties could be manipulated depending on the conditions in which the hydrogels were fabricated. Specifically, the BDDGE-EDC/NHS hydrogels, fabricated by BDDGE coupling followed by carbodiimide chemistry resulted in materials with high elasticity. Interestingly, the slow gelation time of the BDDGE cross-linking may allow for drug encapsulation within the collagen matrix upon fabrication for therapeutic use. In addition, the YIGSR and IKVAV peptide-BDDGE-based hydrogels may also serve as ideal candidates for corneal substitutes given their ability to enhance epithelial and nerve cell growth. 
