# Correction: Koh, L.B., *et al.* Epoxy Cross-Linked Collagen and Collagen-Laminin Peptide Hydrogels as Corneal Substitutes. *J. Funct. Biomater.* 2013, *4*, 162-177

**DOI:** 10.3390/jfb5010027

**Published:** 2014-03-17

**Authors:** Li Buay Koh, Mohammad Mirazul Islam, Debbie Mitra, Christopher W. Noel, Kimberley Merrett, Silvia Odorcic, Per Fagerholm, William Bruce Jackson, Bo Liedberg, Jaywant Phopase, May Griffith

**Affiliations:** 1 Integrative Regenerative Medicine Center, Department of Physics, Chemistry and Biology, Linköping University, SE 581 83 Linköping, Sweden; E-Mails: mayko@ifm.liu.se (L.B.K.); jayph@ifm.liu.se (J.P.); 2 Swedish Nanoscience Center, Karolinska Institute, 171 77 Stockholm, Sweden; E-Mail: mohammad.islam@ki.se; 3 Integrative Regenerative Medicine Center & Department of Clinical and Experimental Medicine, Cell Biology Building, Linköping University, SE 581 85 Linköping, Sweden; E-Mails: kim.merrett@sympatico.ca (K.M.); per.fagerholm@liu.se (P.F.); 4 Ottawa Hospital Research Institute, University of Ottawa Eye Institute, 501 Smyth Rd. Ottawa, ON K1H 8L6, Canada; E-Mails: debbiemitra@hotmail.com (D.M.); christophernoel90@gmail.com (C.W.N.); silvia.odorcic@gmail.com (S.O.); bjackson@ohri.ca (W.B.J.); 5 Center for Biomimetic Sensor Science, Nanyang Technological University, Research Technoplaza, Story 6, 50 Nanyang Drive, Singapore 637553; E-Mail: bliedberg@ntu.edu.sg

It has been brought to our attention very recently that we had an omission error in our methods section of the paper [1]. This is Section 3.4, which should have read as follows: 

The tensile strength, Young’s moduli and elongation at break of the 10% hydrogels were determined on an Instron electromechanical universal tester (Model 3342) equipped with Series IX/S software, using a crosshead speed of 10 mm·min^−1^ and a gauge length for testing of 5 mm. Hydrogels with 0.55 mm thickness were equilibrated in PBS and cut into 10 mm × 5 mm rectangular sheets. The load cell used was 10 N.

For 18% hydrogels, measurements were made on an Instron universal test machine (Biopuls 3343, High Wycombe, UK). The measurements were carried out under water immersion at 37 °C. Dumb-bell shaped hydrogels of 0.5 mm thickness were made for the mechanical properties measurement. The grip area at each end was 6 mm × 10 mm with a gauge segment of 14 mm × 6 mm. The mechanical testing was carried out with the crosshead moving at a speed of 10 mm·min^−1^ and the load cell was 50 N. 

A minimum of three specimens was measured for each hydrogel formulation and repeated for three independent experiments.
